# Genome Sequence Analysis of *Mycoplasma* sp*.* HU2014, Isolated from Tissue Culture

**DOI:** 10.1128/genomeA.01086-15

**Published:** 2015-09-17

**Authors:** Michael J. Calcutt, Bernadett Szikriszt, Ádám Póti, János Molnár, Judit Z. Gervai, Gábor E. Tusnády, Mark F. Foecking, Dávid Szüts

**Affiliations:** a Department of Veterinary Pathobiology, University of Missouri, Columbia, Missouri, USA; b Institute of Enzymology, Research Centre for Natural Sciences, Hungarian Academy of Sciences, Budapest, Hungary

## Abstract

The draft genome sequence of a novel *Mycoplasma* strain, designated *Mycoplasma* sp. HU2014, has been determined. The genome comprises 1,084,927 nucleotides and was obtained from a mycoplasma-infected culture of chicken DT40 cells. Phylogenetic analysis places this taxon in a group comprising the closely related species *Mycoplasma yeatsii* and *Mycoplasma cottewii*.

## GENOME ANNOUNCEMENT

Mycoplasmas, members of the wall-less *Mollicutes* (1), include multiple species that are important pathogens of humans and animals (2). In addition, several species are notorious contaminants of tissue culture (3), necessitating rigorous testing for inadvertent growth that may confound biomedical research or adversely impact biomanufacturing (4). Commonly reported estimates of contamination rates range from 15 to 35%, although incidences of 65 to 80% have been reported for these parasitic blights (5). Consistent with such values, a recent study revealed a high incidence of mycoplasma-derived reads in the NCBI mammalian RNA-seq archive (6).

As part of our ongoing mutagenesis studies in the chicken DT40 bursal lymphoma cell-line (7), whole-genome sequencing disclosed an inadvertent *Mycoplasma* coculture with a DT40 clone. The strain, designated *Mycoplasma* sp. HU2014, was almost identical to *Mycoplasma yeatsii* and *Mycoplasma cottewii* based on 16S rRNA analysis. These serologically distinct species (8) have almost identical 16S rRNA sequences, differing at 4 single nucleotide polymorphisms (9). Contamination with *M. yeatsii*, but not *M. cottewii*, has been reported once (10), and so is extremely rare in comparison with other mycoplasmas. Despite *M. yeatsii* and *M. cottewii* being considered caprine commensals or opportunists (11), the most likely source of the contamination was fetal bovine serum.

To gain further insight into this taxon, Illumina 150-bp paired-end reads were assembled *de novo* (12) and screened by Mega BLASTn to retrieve contigs that matched the complete genomes of *M. yeatsii* or *Mycoplasma putrefaciens*. The resulting 75 contigs were auto-annotated (13) and then manually curated based on the *M. yeatsii* GM274B genome features (14). The final data set comprised 1,084,927 nucleotides (25.4% GC; >6,800-fold average coverage depth), which is larger than that of *M. yeatsii* GM274B (895 kb), indicating that most, if not all, of the genome had been retrieved. A total of 1,080 genes were annotated (including partial genes at contig termini), including 910 open reading frames, 30 tRNAs, and 3 small RNAs.

Based on the available sequence data and published molecular typing schemes, it was not possible to unambiguously assign HU2014 to a known species. Both 16S rRNA-based and multilocus sequence typing indicate very close relationship between *M. cottewii* and *M. yeatsii* (15). The 5 multilocus sequence type targets from HU2014 exhibited 93 to 98% identity to their respective orthologs from these two species. For *rpoB* and *lepA*, the *M. cottewii* and *M. yeatsii* sequences were more similar to each other (98% identity) than either was to HU2014 (95 to 96%), but this pattern did not comport for the other targets. Further comparative analysis is warranted to delineate the fine structure of this cluster of related taxa.

These data provide evidence for a distinct evolutionary history for the HU2014 lineage and suggest future taxonomic refinement to accommodate the spectrum of sequence variation encompassed within this clade. As HU2014 is cytotoxic to DT40 cells, it will be of interest to determine the molecular attributes responsible for pathogenesis. These data also serve as a further precautionary note on the difficulties associated with mycoplasma prevention in eukaryotic culture systems.

### Nucleotide sequence accession number.

This whole-genome shotgun project has been deposited at DDBJ/EMBL/GenBank under accession number LFYS00000000.

## References

[1] Razin S, Yogev D, Naot Y 1998 Molecular biology and pathogenicity of mycoplasmas. Microbiol Mol Biol Rev 62:1094–1156. 9841667 10.1128/mmbr.62.4.1094-1156.1998 PMC98941

[2] Simecka JW, Davis JK, Davidson MK, Ross SE, Städtlander CTKH, Cassell GH 1992 Mycoplasma diseases of animals, p 391–416. *In* Maniloff J, McElhaney RN, Finch LR, Baseman JB (ed), Mycoplasmas: molecular biology and pathogenesis. ASM Press, Washington, DC.

[3] McGarrity GJ, Kotani H, Butler GH 1992 Mycoplasmas and tissue culture cells, p 445–456. *In* Maniloff J, McElhaney RN, Finch LR, Baseman JB (ed), Mycoplasmas: molecular biology and pathogenesis. ASM Press, Washington, DC.

[4] Armstrong SE, Mariano JA, Lundin DJ 2010 The scope of mycoplasma contamination within the biopharmaceutical industry. Biologicals 38:211–213. doi:10.1016/j.biologicals.2010.03.002. 20362237

[5] Drexler HG, Uphoff CC 2002 Mycoplasma contamination of cell cultures: incidence, sources, effects, detection, elimination, prevention. Cytotechnology 39:75–90. doi:10.1023/A:1022913015916. 19003295 PMC3463982

[6] Olarerin-George AO, Hogenesch JB 2015 Assessing the prevalence of mycoplasma contamination in cell culture via a survey of NCBI's RNA-seq archive. Nucleic Acids Res 43:2535–2542. doi:10.1093/nar/gkv136. 25712092 PMC4357728

[7] Molnár J, Póti Á, Pipek O, Krzystanek M, Kanu N, Swanton C, Tusnády GE, Szallasi Z, Csabai I, Szüts D 2014 The genome of the chicken DT40 bursal lymphoma cell line. G3 (Bethesda) 4:2231–2240. doi:10.1534/g3.114.013482. 25227228 PMC4232548

[8] DaMassa AJ, Tully JG, Rose DL, Pitcher D, Leach RH, Cottew GS 1994 *Mycoplasma auris* sp. nov., *Mycoplasma cottewii* sp. nov., and *Mycoplasma yeatsii* sp. nov., new sterol-requiring Mollicutes from the external ear canals of goats. Int J Syst Bacteriol 44:479–484. doi:10.1099/00207713-44-3-479. 8068541

[9] Heldtander M, Pettersson B, Tully JG, Johansson KE 1998 Sequences of the 16S rRNA genes and phylogeny of the goat mycoplasmas *Mycoplasma adleri*, *Mycoplasma auris*, *Mycoplasma cottewii* and *Mycoplasma yeatsii*. Int J Syst Bacteriol 48:263–268. doi:10.1099/00207713-48-1-263. 9542096

[10] Del Giudice RA 1998 M-CMRL, a new axenic medium to replace indicator cell cultures for the isolation of all strains of *Mycoplasma hyorhinis*. In Vitro Cell Dev Biol Anim 34:88–89. doi:10.1007/s11626-998-0087-9. 9542642

[11] DaMassa AJ, Wakenell PS, Brooks DL 1992 Mycoplasmas of goats and sheep. J Vet Diagn Invest 4:101–113. doi:10.1177/104063879200400126. 1554763

[12] Boisvert S, Laviolette F, Corbeil J 2010 Ray: simultaneous assembly of reads from a mix of high-throughput sequencing technologies. J Comput Biol 17:1519–1533. doi:10.1089/cmb.2009.0238. 20958248 PMC3119603

[13] Seemann T 2014 Prokka: rapid prokaryotic genome annotation. BioInformatics 30:2068–2069. doi:10.1093/bioinformatics/btu153. 24642063

[14] Calcutt MJ, Kent BN, Foecking MF 2015 Complete genome sequence of *Mycoplasma yeatsii* strain GM274B (ATCC 43094). Genome Announc 3(2):e00328-15. doi:10.1128/genomeA.00328-15. PMC4408338 25908137

[15] Manso-Silván L, Perrier X, Thiaucourt F 2007 Phylogeny of the *Mycoplasma mycoides* cluster based on analysis of five conserved protein-coding sequences and possible implications for the taxonomy of the group. Int J Syst Evol Microbiol 57:2247–2258. doi:10.1099/ijs.0.64918-0. 17911291

